# Calcium, Vitamin D, and Health

**DOI:** 10.3390/nu12020416

**Published:** 2020-02-06

**Authors:** Luis Gracia-Marco

**Affiliations:** PROFITH “PROmoting FITness and Health through Physical Activity” Research Group, Sport and Health University Research Institute (iMUDS), Department of Physical Education and Sports, Faculty of Sport Sciences, University of Granada, 18071 Granada, Spain; lgracia@ugr.es

Calcium is the main mineral in the body. It is involved in a variety of structural and functional roles, but the maintenance of calcium homeostasis is perhaps the most studied function of vitamin D. This Special Issue of *Nutrients*, “Calcium, Vitamin D, and Health” contains 12 original publications and two reviews investigating the contribution of (mainly) vitamin D and calcium on relevant health outcomes in a variety of populations, which reflect the evolving and broad interests of research on this topic.

Three studies were published examining the association between vitamin D and body composition. Rabufetti et al. [1] observed that an increase in body fat percentage was a risk factor for 25-hydroxyvitamin D [25(OH)D] insufficiency in a healthy population of 1045 late adolescent males living in southern Switzerland. Abboud et al. [2], in their study on men and women with overweight/obese and undergoing a weight loss program, found greater weight loss, as well as a larger reduction in body mass index (BMI), and waist circumference in those with higher baseline 25(OH)D levels. Moreover, a similar effect was also observed in those with insufficient baseline 25(OH)D levels but supplemented with vitamin D3 for three months. In a study with middle-aged sedentary adults, De-la-O et al. [3] found negative associations between 1,25-dihydroxyvitamin D (1,25(OH)2D, also known as calcitriol, and BMI, lean mass index, and bone mineral density (BMD). The latter finding backs up the notion that 1,25(OH)2D increases bone resorption via stimulating intestinal calcium absorption after calcium intake.

Two other studies investigated the links between 25(OH)D and bone outcomes in young populations. Gil-Cosano et al. [4] revealed a mediating effect of muscular fitness on the relationship between 25(OH)D levels and BMD in children who were overweight/obese, while Rapun-Lopez et al. [5] showed similar bone remodeling in adolescent male cyclists than age-matched active controls over one year, but lower 25(OH)D. In adult and older women from the Chilean National Health Survey 2016–2017 (total *N* = 1931), Solis-Urra et al. [6] found a joint association of high sedentary time/passive commuting to be associated with 25(OH)D deficiency, even after controlling for sun exposure. This finding connects with the studies mentioned above [1,2,3] due to the proposed link between sedentary time and increased adiposity, as well as between adiposity and reduced 25(OH)D levels.

Libuda et al. [7] studied six single nucleotide polymorphisms (SNPs), which were genome-wide significantly associated with 25(OH)D concentrations in more than 79,000 subjects from the SUNLIGHT genome-wide association study (GWAS). However, they did not identify the potential role (from a genetics perspective) of 25(OH)D in the onset of depressive symptoms or broad depression. Multiple sclerosis (MS) has been negatively associated with BMD through various factors, and previous research has suggested that vitamin D could play a role in the pathogenesis of MS by possibly modulating T-lymphocyte subset differentiation. In this regard, Vlot et al. [8] studied the vitamin D-fibroblast-growth-factor-23 (FGF23) and measured multiple vitamin D metabolites and bone turnover markers in a cohort of MS patients and healthy controls. They found lower serum concentrations of total 25(OH)D, free 25(OH)D, free 1.25(OH)2D, and 24,25 dihydroxyvitamin D [24,25(OH)2D] in female MS patients compared with their healthy peers, while serum concentrations of vitamin D binding protein (VDBP) were higher in male MS patients, compared with male controls. This study strengthens the idea that a single measurement of total 25(OH)D may not be enough to fully reflect all changes in vitamin D metabolism in MS patients. In a randomized clinical trial conducted in hypertensive adults, Francic et al. [9] did not support the routine measurement of 24,25 dihydroxycholecalciferol (24,25(OH)2D3) in order to individually optimize the dosage of vitamin D supplementation. Interestingly, the activity of 24-hydroxylase increased after vitamin D supplementation. In patients with postherpetic neuralgia (PHN), Chen et al. [10] showed a higher prevalence of hypovitaminosis D (as reflected by 25(OH)D levels) than in the controls, and those with hypovitaminosis D also had a lower vitamin D supplementation rate and greater pain intensity.

In healthy post-menopausal women, Reyes-Garcia et al. [11] investigated the response of serum 25(OH)D and its predictive factors after a 24-month dietary intervention with milk fortified with vitamin D and calcium. It was found that the improvement in 25(OH)D after the intervention was mainly dependent on the baseline levels of serum 25(OH)D and the percentage of body fat. The study by Jurimae et al. [12] is one of the few studies investigating the association between calcium and adiposity in young populations, and found inverse associations between dietary calcium intake and total body and abdominal adiposity in healthy male adolescents. 

Finally, two timely reviews were included in this Special Issue. Brandao-Lima et al. [13] conducted a systematic review of randomized controlled trials aiming to discuss food fortification as a strategy for maintenance or recovery of nutritional status related to vitamin D in children. Marino and Misra [14], in their review, discussed the biological effects of vitamin D beyond the skeleton, using evidence from randomized controlled trials and meta-analyses. 

The present Special Issue provides a short summary of the progress on the topic of calcium, vitamin D, and human health in different populations, which will be of interest from a clinical and public health perspective. It also underlines the current limitations and the necessity of more powerful study designs to further advance in the knowledge. 
